# From antimicrobial to anticancer: the pioneering works of Prof. Luiz Rodolpho Travassos on bioactive peptides

**DOI:** 10.1007/s42770-023-01118-8

**Published:** 2023-09-19

**Authors:** Saara A. Koskela, Carlos R. Figueiredo

**Affiliations:** 1 Medical Immune Oncology Research Group (MIORG), Institute of Biomedicine, Faculty of Medicine, University of Turku, Turku, Finland; 2 InFLAMES Research Flagship Center, University of Turku, Turku, Finland

**Keywords:** Peptides, Antimicrobial, Phage-display, CDR, Antitumor

## Abstract

Prof. Luiz Rodolpho Travassos, a distinguished Brazilian scientist, was instrumental in fostering an interdisciplinary research approach that seamlessly combined microbiology and oncology. This work has opened new pathways into the understanding of tumorigenesis and aided in the development of innovative therapeutic tools. One significant area of his work has been the exploration of bioactive peptides, many of which were first identified for their antimicrobial properties. These peptides demonstrate promise as potential cancer therapeutics due to their selectivity, cost-effectiveness, ease of synthesis, low antigenicity, and excellent tissue penetration. Prof. Travassos’ pioneering work uncovered on the potential of peptides derived from microbiological sources, such as those obtained using phage display techniques. More importantly, in international cooperation, peptides derived from complementarity-determining regions (CDRs) that showed antimicrobial activity against *Candida albicans* further showed to be promising tools with cytotoxic properties against cancer cells. Similarly, peptides derived from natural sources, such as the gomesin peptide, not only had shown antimicrobial properties but could treat cutaneous melanoma in experimental models. These therapeutic tools allowed Prof. Travassos and his group to navigate the intricate landscape of factors and pathways that drive cancer development, including persistent proliferative signaling, evasion of tumor suppressor genes, inhibition of programmed cell death, and cellular immortality. This review examines the mechanisms of action of these peptides, aligning them with the universally recognized hallmarks of cancer, and evaluates their potential as drug candidates. It highlights the crucial need for more selective, microbiology-inspired anti-cancer strategies that spare healthy cells, a challenge that current therapies often struggle to address. By offering a comprehensive assessment of Prof. Travassos’ innovative contributions and a detailed discussion on the increasing importance of microbiology-derived peptides, this review presents an informed and robust perspective on the possible future direction of cancer therapy.

## Introduction

Prof. Luiz R. Travassos was an eminent figure with expertise spanning microbiology, immunochemistry, cellular and molecular immunology, and experimental oncology. Travassos’ contributions have been crucial in expanding our understanding of these fields. His scientific discoveries, honed at esteemed institutions including Columbia University and Memorial Sloan Kettering Cancer Center during the 1970s, have spearheaded innovative research endeavors. Travassos initially established his laboratory in New York to investigate physiology of yeasts and to develop microbiological methods for quaternary ammonium compounds such as carnitine and choline [1–7].

While in the US, Travassos found himself amidst some of the brightest minds in immunology, and his interest in immunochemistry was stirred by Kenneth O. Lloyd and Elvin Kabat’s work on the immunochemistry of carbohydrates and polysaccharides. This led to his substantial contributions to the study of heteropolysaccharides [8–13]. But it was at the Memorial Sloan Kettering Cancer Center where Travassos concentrated his research on cancer. Here, in collaboration with Prof. Lloyd J. Old, known as the father of modern tumor immunology, he identified and classified molecules in cancer patient samples, searching for specific antigens of malignant melanoma cells for immunotherapy [14–17].

This period of collaborative research resulted in the identification of GD3 ganglioside as an overexpressed antigen in melanoma, thereby introducing a new era of cancer targets based on glycolipids for the development of novel immunotherapies [15]. Travassos then transferred his extensive knowledge of cancer immunology to a new generation of Brazilian scientists. This dedicated group aimed to expand our understanding of melanoma immunobiology and to create novel therapies. Their focus centered on peptides with antitumor activity, derived from immunoglobulins and other resources.

Peptides, short chains of amino acids, have emerged as promising molecules with diverse biological activities, including their antimicrobial and antitumor functions. Their unique properties, such as high specificity, low toxicity, and the ability to interact with various cellular targets, make peptides an attractive option for developing targeted therapies against cancer [18].

Over the years, extensive research has been conducted to develop bioactive peptides derived from microbiology approaches, such as phage-display, and different sources, such as protein domains, natural resources, and complementary determining regions (CDRs) from immunoglobulins. These investigations have yielded valuable insights not only into the antimicrobial properties of some of these peptides [19–21] but also into the mechanisms of action of their potential in fighting tumor growth, metastasis, and immune evasion. In this review, we delve into the exciting advancements in the field of peptide-based antitumor therapies, highlighting the groundbreaking findings and contributions of Prof. Travassos and his team in unraveling the therapeutic potential of peptides against cancer.

## Phage display of peptides in oncology

George P. Smith initially introduced phage display in 1985, a remarkable technique used in the field of microbiology that facilitated the selection of peptides or proteins with distinct binding affinities [22]. This technique allows for detailed investigations of protein–ligand interactions [23], helps in mapping receptor and antibody-binding sites [24, 25], and enables the enhancement of protein binding characteristics [26, 27]. A core process of phage display known as “panning” is used to concentrate the phage subpopulations that exhibit superior binding affinity to a target [28].

In the domain of oncology, phage display has become an invaluable tool for identifying tumor-homing peptides. Traditional chemotherapy, despite its intention to selectively target rapidly proliferating tumor cells, often affects also healthy tissues, leading to unwanted side effects [29, 30]. Therefore, the selective targeting of tumor cells remains a critical pursuit in cancer research. Phage display plays a crucial role in this endeavor to discover peptides that specifically target cells, enabling the direct delivery of anti-cancer drugs to tumor sites and enhancing treatment selectivity [29, 31].

A significant breakthrough in this research occurred with the groundbreaking study conducted by Pasqualini et al. in 1996 [32]. They introduced an in vivo screening protocol to identify organ-specific targeting peptides, leading to the discovery of peptides homing to specific organs such as the brain, kidney, and blood vessels. Their subsequent work used in vivo phage display to identify tumor-homing peptides and revealed peptide motifs such as Arg-Gly-Asp (RGD) and CDCRGDCFC (RGD-4C) that bind selectively to tumor types and integrins αvβ3 and αvβ5 [33]. Other identified peptide motifs included Asn-Gly-Arg (NGR) targeting aminopeptidase N in tumor vasculature [34] and Gly-Ser-Leu (GSL), found in various tumors [33].

Further expanding upon this work, researchers discovered that solid tumors, as a consequence of their rapid growth, generate their own vasculature through a process known as angiogenesis to fulfill their increased demand for nutrients and oxygen [35]. Phage display techniques were employed by Hetian et al. to identify peptide K237 (HTMYYHHYQHHL), which demonstrates binding affinity to the KDR/Flk-1 receptor of vascular endothelial growth factor (VEGF), a crucial factor involved in angiogenesis. This peptide effectively disrupts the interaction between VEGF and its receptor, resulting in the downregulation of the VEGF signaling pathway. Notably, K237 acts as an anti-angiogenic peptide when tested in vivo [36]. Research teams have also investigated the importance of basic fibroblast growth factor (bFGF) in promoting tumor angiogenesis using phage display. Wu et al. [37] identified P7 peptide (PLLQATL), an antagonist to bFGF, using a phage-displayed heptapeptide library. This peptide inhibited bFGF-induced cell proliferation and angiogenesis.

Also Professor Travassos’ research group made significant contributions to the field of phage display. Matsuo et al. identified a peptide 20 (CSSRTMHHC), which not only delayed tumor growth but also decreased the number of nodules in an experimental melanoma model [38]. In vitro assays showed that peptide 20 could inhibit the tumor cell-to-cell contact, hence interfering with tumor cell establishment. It was further showed that peptide 20 binds to cadherin, a key molecule in the cell–cell adhesions. Alterations in the expression patterns of cadherins are associated with epithelial-mesenchymal transition and subsequent tumor invasion and metastasis [39].

**Fig. 1:**
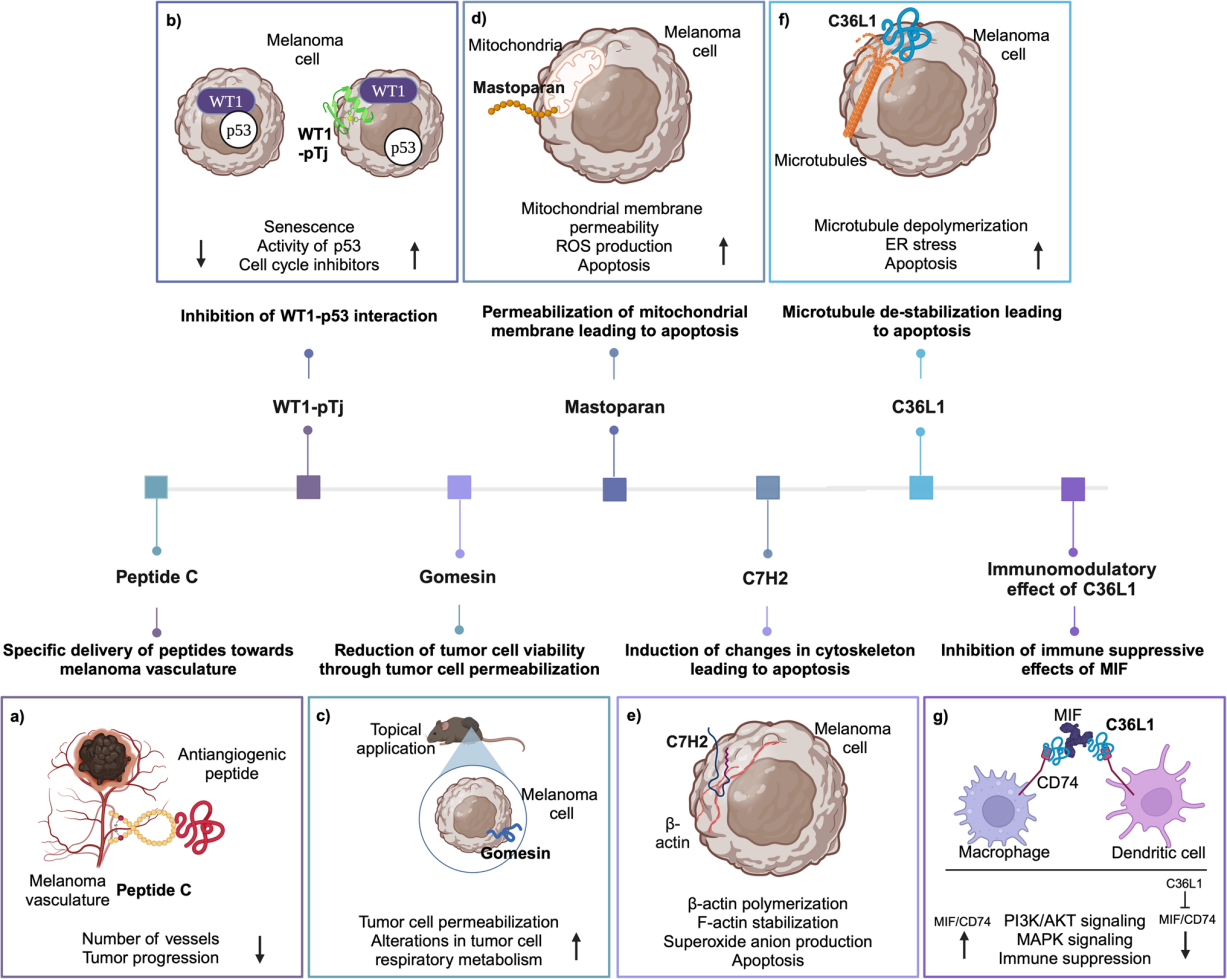
Schematic showing the groundbreaking research of Prof. Travassos and his team. **a** Travassos and his team discovered the melanoma-homing peptide C that was combined with antiangiogenic peptide to specifically target melanoma vasculature. The combination successfully reduced the number of vessels and inhibited tumor progression. **b** WT1-pTj peptide emerged as candidate for therapy when its abilities in enhancing p53 activity and inducing senescence and activity of cell cycle inhibitors in melanoma were discovered. **c** In 2008, the team discovered gomesin, a peptide derived from spider *Acanthoscurria gomesiana*. Topical application of gomesin incorporated in a cream base resulted in tumor cell permeabilization and alterations in tumor cell respiratory metabolism leading to tumor cell death. **d** Another peptide derived from natural sources was mastoparan which exhibited the ability to permeabilize mitochondrial membrane, increase ROS production, and induce apoptosis. **e** C7H2 peptide discovered in 2012 potently induced changes in cytoskeleton such as β-actin polymerization and F-actin stabilization which ultimately led to apoptosis of melanoma cells. **f** The discoveries of Travassos and his team further expanded to the investigation of complementary determining region (CDR)-derived peptides. From these works, C36L1, and microtubule depolymerization-inducing peptide, was identified. **g** Later on, the immunomodulatory properties of C36L1 were discovered as its binding to CD74 disrupted immunosuppressive MIF signaling in macrophages and dendritic cells. Created with BioRender.com

In a subsequential study, Prof. Travassos’ group discovered a novel bioactive antitumor peptide (-CVNHPAFAC), also known as peptide C (Fig. 1a), using a C7C phage-display library that was enriched towards targets on developing tumors in syngeneic mouse models in an in vivo biopanning procedure [40]. The researchers found that the phages displaying peptide C had a pronounced binding preference to melanoma cells in vivo, exhibiting a binding affinity roughly 50 times greater than that of normal tissue. Furthermore, it was observed that a synthesized version of the peptide could effectively displace its corresponding phage from the tumor site. Interestingly, in cell-based enzyme-linked immunosorbent assay (ELISA) tests, the peptide was found to bind more readily to endothelial cells than to melanoma cells thus indicating that the peptide is likely targeting the vasculature of the melanoma. Additionally, the peptide demonstrated an ability to bind to the human sonic hedgehog, a protein known to play a role in the tumorigenesis and progression of various types of human cancers [41]. Building upon this discovery, the researchers further developed a novel peptide therapeutic strategy [40]. By utilizing a GYG linker, they conjugated the cyclic peptide to HTMYYHHYQHHL-NH(2), a recognized antagonist of the VEGFR-2 receptor. As a result, the complete peptide CVNHPAFACGYGHTMYYHHYQHHL-NH(2) was generated. When this peptide was administered systemically, it effectively delayed tumor growth and improved survival, whereas a scrambled homing peptide conjugated with the same antagonist sequence had no observable effect.

This study represents the first report of the successful synthesis of a tumor-homing peptide combined with an antiangiogenic peptide, heralding a promising new direction in the development of anticancer therapeutics.

The advent of phage display marked a significant breakthrough in oncology, refining our understanding of protein–ligand interactions and building the grounds for targeted cancer therapies. Among the notable contributors in this field, Professor Travassos’ group stands out for their pioneering work on tumor-homing peptides. The culmination of their efforts was the innovative synthesis of a tumor-homing peptide linked to an antiangiogenic peptide. This strategy improved the selectivity and efficacy of anticancer therapeutics, representing a significant leap forward in the field. Despite these advances, the journey from laboratory discovery to clinical application remains challenging. The future of this field lies in building upon these foundational studies, further exploring the potential of phage display for the identification of new peptides and refining the development of targeted anticancer therapeutics.

## Antitumor activity based on protein domains

Peptides derived from protein domains, which are specific functional or structural units within proteins, often conserve binding and functional properties. For instance, cathelicidins, a family of antimicrobial peptides, are derived from the cathelin domain of cathelicidin proteins, including the well-recognized LL-37 peptide with antimicrobial activities against numerous gram-negative and gram-positive bacteria [42], and recently repurposed for anticancer therapies [43]. Similarly, defensins, with members such as HBD-1 and HBD-2, are extracted from defensin domains found in an array of proteins with antimicrobial activities [42], have also been pointed to hold antitumor properties [44].

Peptides derived from protein domains play a crucial role in mapping protein–protein interactions and have significant potential in the design of specific binding peptides for target proteins. The characterization of these interactions through methods such as x-ray crystallography or nuclear magnetic resonance is integral to improve the understanding of intermolecular contacts and folding requirements [45]. The landscape of these interactions, known as the interactome, underpins cellular functions and biological networks, with implications in diseases such as cancer.

In line with this rationale for peptide discovery, a study by Prof Travassos’ research group provides a noteworthy contribution to the field. They explored the therapeutic potential of targeting the Wilms tumor protein 1 (WT1) transcription factor in malignant melanoma (Fig. 1b). WT1 has been implicated in cell survival and metastasis, highlighting its potential as a therapeutic target. Their research focused on a lysine-arginine rich peptide, WT1-pTj, derived from WT1’s zinc finger domain, evaluating its potential to work as a vaccination strategy in harnessing an antitumor-specific immune response against melanoma. Surprisingly, the group found that the peptide could exert a direct effect on both human and mouse melanoma cell proliferation [46]. The WT1-pTj peptide exhibited quick cellular penetration and induced senescence, evidenced by an increase in SA-β-galactosidase activity, enhanced p53 transcriptional activity, and induction of the cell cycle inhibitors p21 and p27, reminiscent of the effects seen in previous work on p21WAF1-derived peptides [47]. The WT1-derived peptide, WT1-pTj, has displayed significant effects on cancer cells. It demonstrated the ability to bind to p53, competing with the WT1 protein for binding sites, mirroring the action of the p16-derived peptide with CDK4 and CDK6 [46]. It showed sustainable inhibition of cell growth, the disruption of cell clonogenicity, and initiation of G2/M cell cycle arrest [46]. Beyond in vitro results, WT1-pTj also revealed effectiveness in the in vivo context, curbing both metastatic and subcutaneous growth of murine melanoma in syngeneic mice, and enhancing survival rates in nude mice with human melanoma cells. This 27-amino acid peptide, reliant on C(3) and H(16), displayed its potential as an anti-melanoma agent by inhibiting the proliferation of WT1-expressing human tumor cell lines.

While the WT1 peptide, derived from functional protein domains, has shown substantial potential as a cytotoxic agent against tumor cells in Prof. Travassos’ pioneering work, its antimicrobial properties remain unexplored. Given that peptides from similar origins have demonstrated dual antimicrobial and antitumor properties, there is a strong case for examining the WT1 peptide’s potential as an antimicrobial agent. Hence, Prof. Travassos’ findings open up an exciting avenue for the discovery of novel antimicrobial agents, broadening the impact of his research beyond oncology.

## Bioactive antitumor natural peptides

Bioactive peptides of natural origin can be discovered in diverse sources and, in many cases, are cryptic within the framework of proteins. In this regard, they bear resemblance to the active domains found in various molecules of innate immunity, which have undergone centuries of evolutionary development. Natural peptides continue to be a fertile source of investigation of anticancer agents with a broad variety of structures and different mechanisms of action. Prof Travassos’ group has also shed light on the potential of peptides from natural resources in cancer therapy. One such example is gomesin, a potent antimicrobial peptide (AMP) isolated from the spider *Acanthoscurria gomesiana* (Fig. 1c) [21].

The objective of the research was to explore the antitumor properties of gomesin through both in vitro and in vivo. The study demonstrated that topical application of gomesin, incorporated in a cream base, significantly delayed tumor growth in a subcutaneous murine melanoma model. In vitro experiments further revealed direct cytotoxic effects of gomesin on murine melanoma cells and various human tumor cell lines, with IC(50) values below 5 µM. The optimal activity of gomesin was found to rely on its distinctive beta-hairpin structure, which encompasses disulfide bridges. Interestingly, gomesin also exhibited a membrane-permeabilizing activity, binding to the cell membrane and causing the extracellular release of cytoplasmic lactate dehydrogenase. Furthermore, gomesin facilitated the internalization of macromolecules, such as immunoglobulins, which enhanced its cytotoxic effect. Notably, gomesin also demonstrated a cytotoxic effect on endothelial cells, suggesting its potential as a topical agent for the treatment of intradermal and epithelial skin cancers.

In addition to gomesin, another notable study supervised by Prof Travassos explores the potential of mastoparan, an α-helical and amphipathic tetradecapeptide derived from the venom of the wasp *Vespula lewisii* [48]. Mastoparan has shown initial antimicrobial properties, and was further evaluated for its potential therapeutic agent in cancer treatment, particularly against malignant melanoma (Fig. 1d) [49]. Prof Travassos’ group conducted a study investigating mastoparan’s effects on melanoma using the B16F10-Nex2 murine model and in vitro experiments, revealing its ability to induce melanoma cell death through the mitochondrial apoptosis pathway. Mastoparan exhibited potent cytotoxicity against various cancer cell lines, with a particular efficacy against melanoma cells, suggesting its potential as an anticancer agent. Its mechanism of action involved ROS-dependent oxidative stress generation, selectively targeting cancer cells due to their heightened sensitivity to oxidative stress. Mastoparan permeabilizes cell and mitochondrial membranes, leading to mitochondrial membrane depolarization, increased ROS generation, release of pro-apoptotic proteins, and caspase-dependent apoptosis. In vivo, mastoparan significantly reduced subcutaneous melanoma growth in mice and increased their survival rate, demonstrating its antitumor activity.

The discovery of gomesin and mastoparan and the understanding of their mechanism of action mark the successful use of natural peptides as innovative therapeutic agents against cancer for topical applications, when surgery is not optimal.

## Using the CDRs from immunoglobulins to discover new therapeutic peptides

The complexity of antibody structures, including hypervariable domains resulting from gene recombination and mutations, plays a crucial role in recognition of diverse antigens. Traditionally, the remaining amino acid sequences in the constant regions were believed to possess supportive or functional attributes, without direct anti-infective or anti-tumor activities. However, researchers have now realized the immense potential of different regions of immunoglobulins in developing and optimizing novel therapeutic compounds to combat various diseases.

Of particular interest are the complementary determining regions (CDRs) found in these proteins, as they are responsible for the specific recognition and binding of different target antigens. To identify potential therapeutic peptides, scientists can examine the CDRs of immunoglobulins (Ig) using Cotia’s and Kabat’s rules, which offer a framework for identifying these regions. By analyzing the amino acid sequences within these regions, researchers can gain insights into the biological functions of these peptides, including their antimicrobial and antitumor properties.

Polonelli et al. mapped the active sequence in an anti-idiotypic antibody that displayed cytotoxic effects similar to the killer toxin from *Pichia anomala*, the original immunogen [50]. They found that even peptides derived from the framework sequences flanking the hypervariable complementarity determining regions (CDRs) exhibited cytotoxicity against *Candida albicans* [51]. Further study identified a specific peptide, consisting of three amino acids from VLCDR1 (L1) and seven amino acids from the framework sequence, which was termed the killer peptide (KP) [52]. An engineered derivative of KP, with one substitution on the N-terminal (A1E), showed increased effectiveness and displayed cytotoxic activity against various fungi, protozoa, bacteria, and viruses [50, 53–57]. These findings led to the hypothesis that internal sequences of immunoglobulins (Igs), regardless of antibody specificity, could exhibit anti-infective activities similar to the molecules of innate immunity.

To further expand these studies, Polonelli et al. demonstrated that synthetic peptides, with sequences identical to fragments of the constant region (Fc region) found in IgG, IgM, and IgA classes, were cytotoxic to *Candida albicans, Candida glabrata, Cryptococcus neoformans,* and *Malassezia furfur* [58]. To delve deeper into these discoveries, the researchers focused on the CDRs of monoclonal antibodies (mAbs) with various specificities and tested corresponding synthetic peptides against *Candida*, HIV, and murine melanoma (B16F10). Among the tested peptides, the VH CDR2 (H2) from mAb C7, which targeted a mannoprotein from *C. albicans*, and the VL CDR1 (L1) from mAb HuA, directed against human blood group A, were identified as C-amidated synthetic peptides that induced apoptosis in melanoma cells. Furthermore, these peptides showed protective effects in a metastatic syngeneic model using intravenously challenged C57BL6 mice [19].

Further advances were conducted by Dobroff et al. as they explored the antimicrobial, antiviral, and antitumor activities of monoclonal antibodies and synthetic CDR-related peptides [59, 60]. These peptides exhibited inhibitory activities against microorganisms and cancer cells in vitro, ex vivo, and in vivo, suggesting their potential for the development of therapeutic agents. Monoclonal antibody A4, raised against B16F10 cells, induced morphological changes such as cell blebbing, shrinkage, and loss of adherence as well as apoptosis in three different melanoma cell lines in vitro. Furthermore, the researchers observed complete protection against tumor growth upon mAb A4 treatment in subcutaneously B16F10-Nex2 -challenged mice. The target of A4 on murine melanoma was identified as protocadherin β13, that belongs to the cadherin family related with cell adhesion functions [39]. The study also focused on the VH CDR3 (H3) of A4, which exhibited distinctive properties and competitive binding with the original monoclonal antibody (mAb) against the antigen. Synthetic peptides representing linear and cyclic variants of the A4 H3 region were synthesized and evaluated for their microantibody properties. The linear A4 H3 peptide demonstrated inhibitory effects on melanoma cells and induced DNA degradation, whereas the cyclic extended form displayed reduced efficacy in competing with A4 for binding to tumor cells [60].

In the case of A4M, an IgM antibody, it displayed in vivo activity against metastatic melanoma but not in vitro. A4M reacted with histone-1 in the nuclei of B16F10-Nex2 cells, potentially enhancing the inflammatory response against necrotic tumor cells. Furthermore, three bioactive peptides were generated from the CDRs of A4M. Among them, the VH CDR3 peptide displayed competitive binding with A4M for melanoma cell interaction but did not exhibit cytotoxicity in vitro. On the other hand, CDRs L1 and L2 of A4M inhibited the growth of B16F10-Nex2 cells, induced DNA degradation in melanoma and HL-60 cells, and demonstrated anti-angiogenic effects on human umbilical vein endothelial cells (HUVECs). The biological activities of L1 and L2 were dependent on their specific sequences, as replacing certain amino acids abrogated their activities [60].

Travassos and his team also discovered the peptide C7H2, which triggers tumor apoptosis and reduces melanoma growth (Fig. 1e) [19]. Subsequent research by Arruda et al. [61] demonstrated the apoptotic effect of C7H2 extended to various human tumor cell lines at similar EC50 concentrations, suggesting a common target. The binding of biotinylated-C7H2 to B16F10-Nex2 cell extract identified β-actin as the primary ligand. C7H2 induced polymerization of G-actin and enhanced stabilization of F-actin resulting in initiation of apoptosis and abundant production of superoxide anions. Peptide binding and internalization activated caspases 3 and 8, resulting in chromatin condensation, nuclear lamin disruption, DNA degradation, and phosphatidylserine translocation. Similar effects were observed also in syngeneic murine melanoma model of B16F10-Nex2 cells as the administration of C7H2 resulted in significantly reduced number of lung metastases [61].

The exploration of new peptides with antitumor activity through this type of research unveils previously undisclosed molecular mechanisms underlying the interactions between cancer cells and the immune system. The studies also highlighted that immunoglobulin (Ig) molecules provide an unlimited source of sequences for therapeutic development. As a result, it enhances our understanding of their functional interplay.

## CDR-derived antitumor peptides with immunostimulatory properties

Building upon the conception of discovering antitumor peptides from Ig-CDRs, Travassos’ team advanced their research by applying immunostimulatory screening tests to develop new immunotherapeutic antitumor peptides. In this context, the study conducted by Figueiredo et al. [62] stands as a significant milestone. The study aimed to identify CDR-derived peptide sequences with potent antitumor activities, both in vitro and in vivo, while also possessing immunostimulatory properties by stimulating macrophages in vitro [63]. By investigating short synthetic peptides corresponding to conserved CDR sequences from various immunoglobulin families, promising candidates for immunotherapy were discovered. These peptides exhibited cytotoxic effects against murine melanoma cells and a range of human tumor cell lines in in vitro experiments. Additionally, they demonstrated remarkable anti-metastatic effects in a syngeneic melanoma model in mice.

Next, Figueiredo et al. proceeded to evaluate the antitumor properties of one of these peptides, the C36L1 peptide, which demonstrated both immunostimulatory properties and direct antitumor activities selectively against melanoma cells, both in vitro and in vivo (Fig. 1f) [64]. Mechanistically, C36L1, derived from the light-chain CDR1 sequence, directly inhibits melanoma cells by inducing microtubule depolymerization and apoptosis. It also hinders migration, invasion, and proliferation by regulating the PI3K/Akt signaling axis. In vivo, C36L1 delays tumor growth, elicits immune response-dependent anti-tumor protection, and demonstrates efficacy when dendritic cells stimulated ex vivo with the peptide are transferred to tumor-challenged animals. The C36 VL CDR1 peptide shows promise as a microtubule-interacting drug, inducing tumor cell death through apoptosis and inhibiting metastases of aggressive melanoma cells.

The results obtained with dendritic cells stimulated ex vivo by the peptide required further evaluation from a mechanistic standpoint to gain deeper insights into the underlying mechanisms by which melanoma cells potentially inhibit immunogenicity of dendritic cells. Figueiredo et al. demonstrate that C36L1’s systemic ability to induce antitumorigenic responses is immune dependent [65]. Mechanistically, the peptide restores immunogenic functions in DCs by binding CD74 on the cells surface, disrupting its interaction with the tumor-derived macrophage inhibitory factor (MIF) and the immunosuppressive signaling consequent of this interaction (Fig. 1g). These groundbreaking findings provided a rationale for further development of peptide-based immunotherapies targeting MIF-CD74 signaling to restore the antitumor immune response in metastatic melanoma. Building upon this, Azevedo et al. combined immune checkpoint therapies with MIF inhibitors to overcome resistance to immune checkpoint blockade (ICB) therapy in melanoma [66]. Their study revealed that inhibition of the MIF blockade in combination with ipilimumab (anti-CTLA-4) improved the response to anti-CTLA-4 treatment in resistant melanoma. The combined therapy enhanced CD8 + T-cell infiltration, facilitated M1 conversion of macrophages, and reprogrammed the metabolic pathway of melanoma cells. High expression of MIF was found to correlate with poor response to ICB therapy in melanoma patients, supporting the potential of combining anti-CTLA-4 with MIF inhibitors as a strategy to enhance ICB therapy response and promote innate immunity activation, metabolic reprogramming, and reduced PD-L1 expression in melanoma cells.

The pioneering research conducted by Prof Travassos and his team in isolating CDRs and harnessing their potential to generate highly specific peptides has revolutionized the field of cancer immunotherapy. The discovery of these peptides with potent antimicrobial and antitumor properties has opened up exciting new avenues for therapeutic development. The remarkable biological functions exhibited by these peptides have surpassed the traditional scope of antibodies, offering novel possibilities for targeted treatments. As researchers continue to unravel the secrets of these fascinating molecules, the field of immunology remains a fertile ground for further exploration and advancements.

## Conclusions

In conclusion, Prof. Travassos and his team have spearheaded an innovative intersection of microbiology and oncology, revealing the immense therapeutic potential of antimicrobial peptides in cancer treatment as illustrated in Fig. 1. Leveraging advanced microbiology techniques like phage display, they have identified tumor-homing peptides with extraordinary binding capabilities, reshaping the understanding of protein–ligand dynamics. Their exploration into peptides derived from protein domains, such as WT1-pTj, alongside naturally sourced ones, such as gomesin and mastoparan, has underscored the impressive antitumor activities of these molecules. The team’s deep dive into CDR-derived peptides, particularly C36L1, has shed light on complex immunostimulatory properties and the mechanisms that allow melanoma to evade the immune system. This groundbreaking work facilitates the development of strategic combination therapies to enhance the efficacy of immune checkpoint interventions, providing targeted solutions to cancer’s inherent challenges. Prof. Travassos’ pioneering contributions continue to drive the evolution of peptide-based cancer therapies and affirm the pivotal role of interdisciplinary research in the advancement of microbiology and oncology.
